# Effects of Doping Elements on the Friction and Wear of SUJ2 Steel Sliding against Aluminum Alloys

**DOI:** 10.3390/mi8040096

**Published:** 2017-03-23

**Authors:** Yuh-Ping Chang, Zi-Wei Huang, Huann-Ming Chou

**Affiliations:** Department of Mechanical Engineering, Kun Shan University, Tainan 710, Taiwan; g0910799199@gmail.com (Z.-W.H.); hmchou@mail.ksu.edu.tw (H.-M.C.)

**Keywords:** aluminum alloy, silicon, friction, wear

## Abstract

Damage to mechanical components caused by wear is considered to be an important issue for mechanical engineers. For the purpose of wear resistance, it is necessary to improve the material properties of the mechanical elements. Furthermore, low friction plays an important role in saving energy. Hence, it is important to establish a key technology for wear resistance and low friction through appropriate materials science for related industries. In general, the tribological properties of aluminum alloys are very different from those of steels. Hence, aluminum alloys should be specially considered and clarified for their tribological properties before being applied industrially. This paper therefore aims to further investigate the effects of the content of doping elements on the friction and wear of the selected aluminum alloys. From the experimental results, it can be concluded that the higher the Si content, the smaller the friction coefficient, and the milder the variation. The higher the content of iron and copper, the more materials are removed, showing better machinability. Moreover, three frictional models and wear mechanisms that describe the effects of the content of doping elements on the friction and wear are proposed. The wear mechanisms change as the silicon content increases, from the junction growth to the wedge and the ploughing particles. As a result, better choices of aluminum alloys with regards to friction and wear can then be made. These results have great practical importance.

## 1. Introduction

With the rapid developments of traffic vehicles and key transmission components, the demand for safe and lightweight new materials is growing, and with the global demand for energy saving and carbon reduction, the industry urgently needs to develop lightweight, reliable and environmentally friendly materials. Of the metal contained in the earth, aluminum is the second most abundant, behind iron. Hence, aluminum and its chemical compositions are the materials with large development potential [1,2,3,4]. In addition, due to its light weight, high strength, acceptable corrosion resistance and good machinability, aluminum alloy is widely used in aerospace machinery [5], defense and people’s livelihood industry. The early aluminum alloy material aimed at being lightweight, and did not consider the enhancement of wear resistance, therefore, although aluminum alloy had high strength and durability, its abrasion resistance was always significantly worse than that of steel [6]. In order to apply aluminum alloy to advanced transport vehicles and key transmission parts, further improvement of the wear properties of aluminum alloy materials is the main project of product development.

Since silicon is a very strong element, if its particles are added to the industrial material, the abrasion resistance of the material can be greatly enhanced, and therefore its applicability is greatly increased [7]. Sarkar and Prasad [8,9] found that the crystal structures of silicon will affect the wear properties of aluminum alloys. If they are further mixed with an appropriate amount of Ni, Cu and Mg, their mechanical properties can then be improved. Over recent decades, these research topics have been continuously investigated by national scholars [10,11,12,13,14,15,16].

Some researchers have found that certain elements may degrade wear properties such as Fe or Mn. It is generally believed that this is because Fe and Mn react with silicon to produce some special compounds, which reduce the effective volume of the silicon particles and reduce the wear properties [17]. Wear behavior refers to the gradual removal of solid surface material by mechanical actions; if the wear rate is lower, a longer experimental time is required to identify the difference [18]. Moreover, Alpas, Hu, and Zhang [19,20,21], Li and Tandon [22], and Subramanian [23] designed dry wear tests for various aluminum alloys to speed up the experiments and obtain clearer results. Wang [24,25] clarified the relations between the frictional temperature, wear, microstructures, normal loads and sliding speeds of the steel 52100. The studies of Moghadam [26,27,28] revealed that the addition of suitable elements to metals decreases both the friction coefficients and wear rate as well as increasing the tensile strength. Moreover, the composites have good tribological properties under limited lubricated conditions due to graphite particles acting as a solid lubricant on worn surfaces.

Based on the above documents, five aluminum alloys which are frequently used in industrial circles are used in this study for friction and wear experiments, in order to analyze and compare the effects of doping elements on the friction and wear of SUJ2 steel sliding against the aluminum alloys under the selected experimental conditions.

## 2. Experimental Apparatus and Procedures

**Figure 1:**
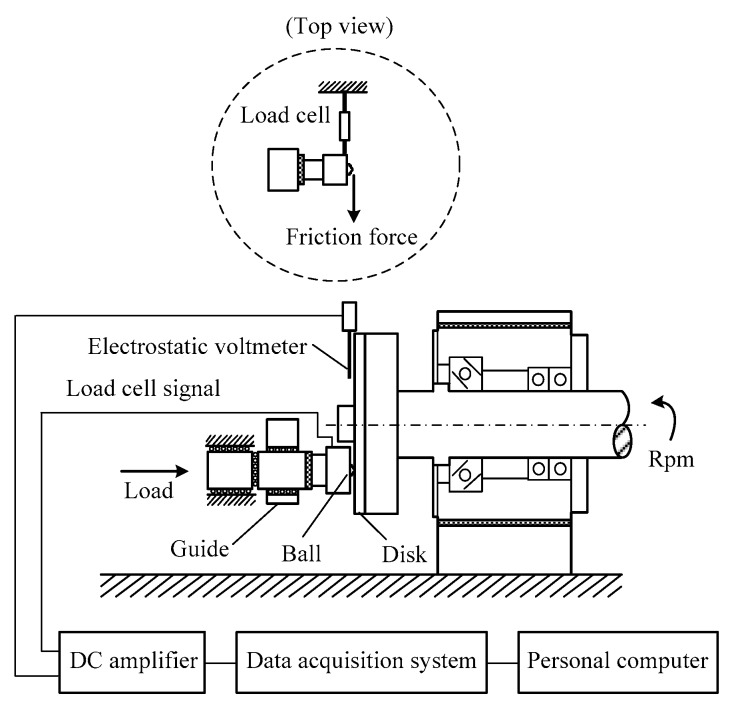
Schematic diagram of the ball/disk friction tester with the measuring system.

Figure 1 shows a schematic diagram of the ball/disk friction tester and the measurement system. The friction coefficient is dynamically measured and exported by variations of electrical voltage through the load cell. The electronic signals are captured by a data acquisition system, and these data are processed and analyzed by a personal computer. Wear loss is measured and quantitatively analyzed by a microbalance, and microscopic wear particles are qualitatively observed by the SEM.

**Figure 2:**
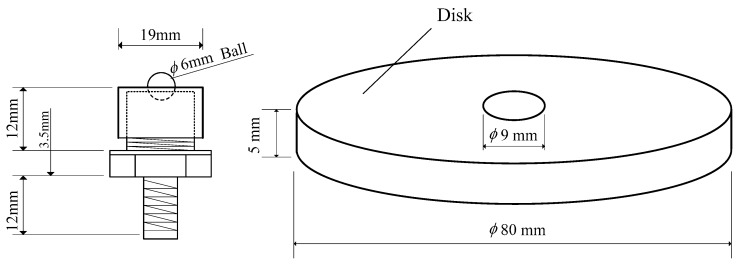
Size and shape of the specimens.

The SUJ2 steel ball is used as the Pin, and the five aluminum alloys are alternately used as the Disk, in the order of 1050, 5052, 5083, 6061 and 7075 aluminum alloys. The contents of the five disks are shown in Table 1. The size and geometry of the test specimens are shown in Figure 2. The sliding distance is set to 100 m, the load is set to 30 N and 60 N, respectively, and the sliding speed is 200 mm/s in this study. The response time of this measurement system is less than 1 ms and the accuracy is 0.1% of the full scale. The experiments were carried out under dry friction conditions. Room temperature was maintained at 25 ± 2 °C, and relative humidity was maintained at 70 ± 5%.

## 3. Results and Discussion

### 3.1. Friction Coefficient

**Figure 3:**
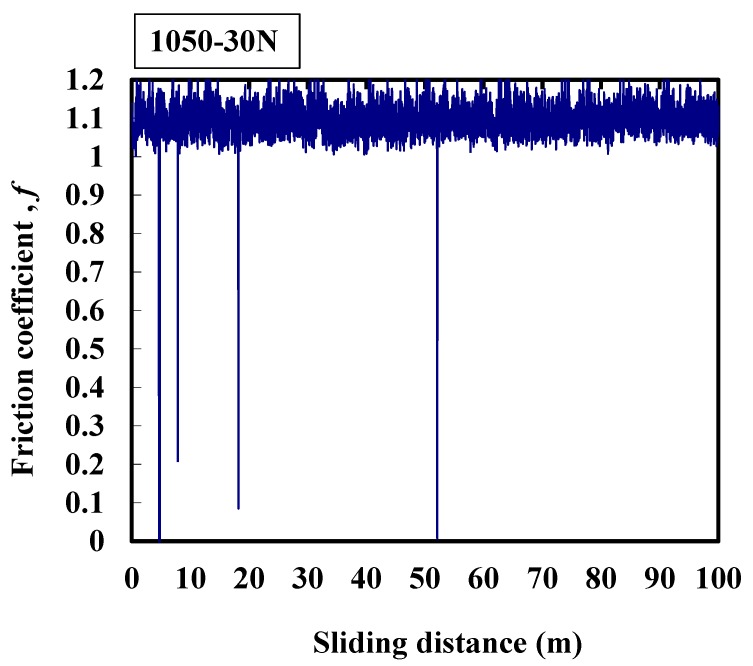
Typical response of the friction coefficient of 1050 alloy at 30 N.

**Figure 4:**
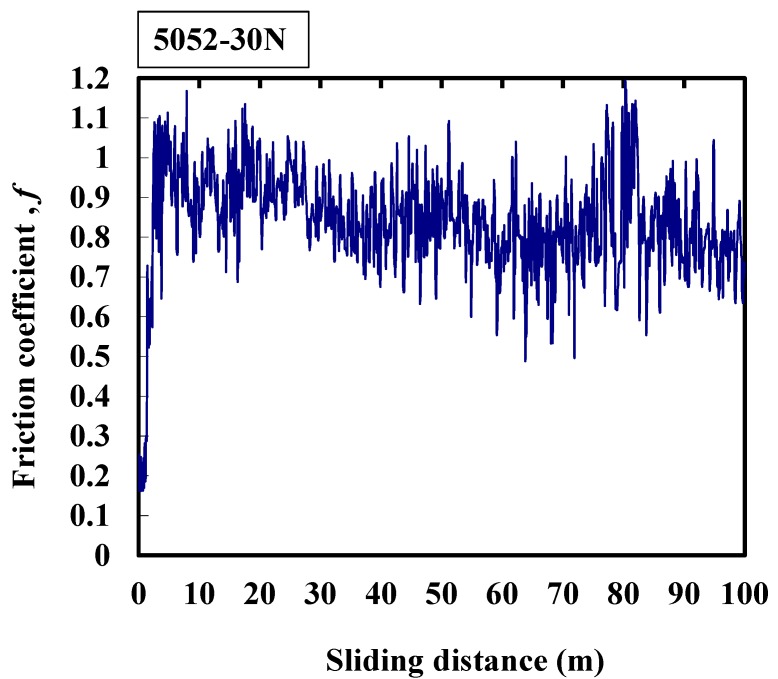
Typical response of the friction coefficient of 5052 alloy at 30 N.

**Figure 5:**
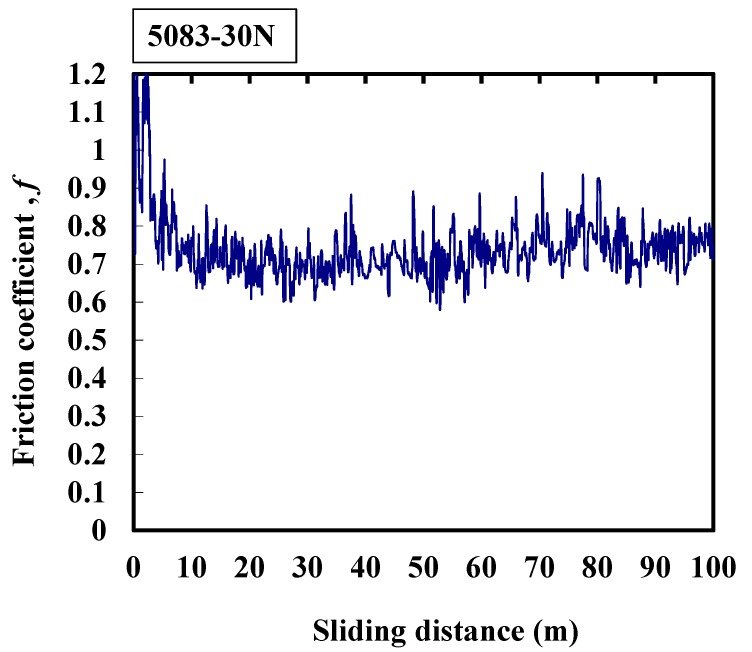
Typical response of the friction coefficient of 5083 alloy at 30 N.

**Figure 6:**
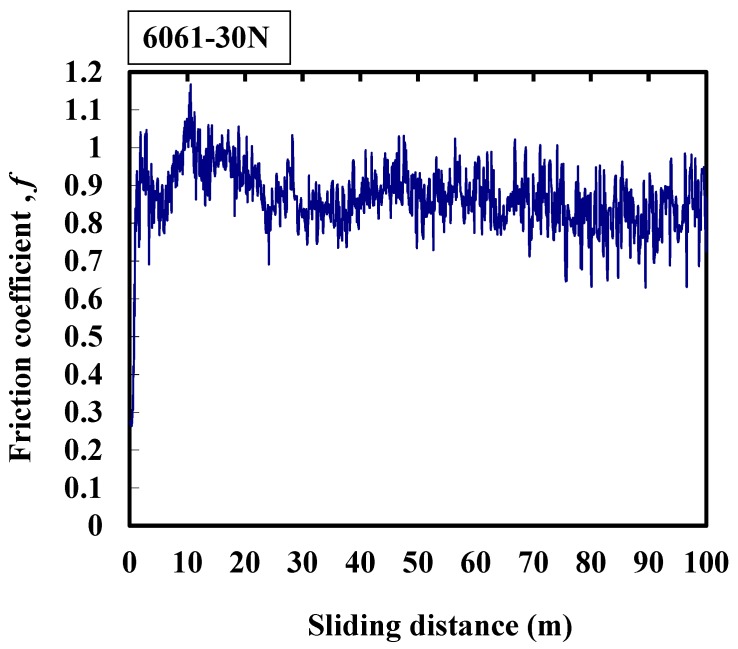
Typical response of the friction coefficient of 6061 alloy at 30 N.

**Figure 7:**
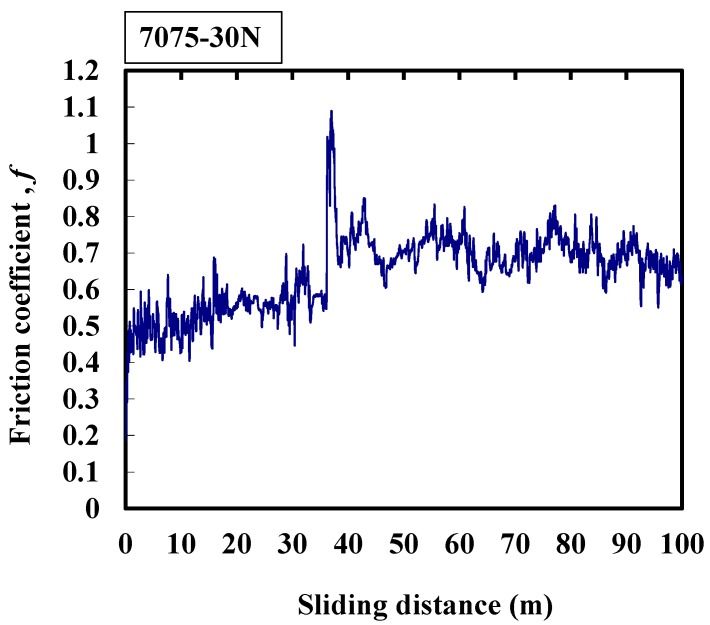
Typical response of the friction coefficient of 7075 alloy at 30 N.

Three reproducibility experiments were conducted. The typical results are shown in Figure 3, Figure 4, Figure 5, Figure 6 and Figure 7. Figure 3 shows that the average friction coefficient of 1050 is 1.1. Generally, the friction coefficient changes in the range of 1.0 to 1.2, the amplitude is large, and the friction frequency is high. Occasionally, during the experimental process, the friction coefficient suddenly decreases to 0, which may indicate the jitter between the interfaces resulting from the wear particles. The oxidation film has broken into small particles between the contact interfaces, and consequently there are some variations between the frictional interfaces. The vibration amplitude of the machine is larger during the experimental process.

Figure 4 shows that the average friction coefficient of 5052 aluminum alloy is about 0.85. At the beginning of the friction process, the friction coefficient increases from 0.15, and the maximum friction coefficient appears at a sliding distance of 80 m. The large amplitude of the friction coefficient means that the friction is very severe, and because of the sharp change in the friction coefficient, it matches the large vibration amplitude of the machine during the experiment.

The average friction coefficient of 5083 is about 0.73 as shown in Figure 5. At the beginning of the friction process, the friction coefficient increases from 0.9 to 1.2. The friction coefficient swings at around 0.7 after the sliding distance of 5 m. Moreover, it shows a large amplitude and high friction frequency which indicate that the oxidation film has broken into small particles between the contact interfaces. As a result, the vibration amplitude of the machine is larger during the experimental process.

The friction coefficient shown in Figure 6 is between that in Figure 4 and Figure 5; it is representative of a few exceptions in the series. It shows that the average friction coefficient of 6061 aluminum alloy is about 0.9, the amplitude is large and the friction frequency is high. During the experimental process, the friction coefficient value changes very drastically. This may indicate the jitter between the interfaces resulting from the wear particles. Moreover, the temperature rises during the friction process [24,25], and the material is therefore softened, resulting in adhesion; and the softened surface is ploughed, generating a large and long slip tongue.

The average friction coefficient of 7075 aluminum alloy is about 0.7 as shown in Figure 7. At the beginning of the friction process, the friction coefficient increases from 0.5, and the maximum of friction coefficient appears at a sliding distance of 38 m. This may indicate that the temperature rises during the friction process, the material is therefore softened, resulting in adhesion, and low frequency wear arises; and the softened surface is ploughed, generating a large and long slip tongue. As a result, the response frequency of the friction coefficient is low. On the other hand, the friction coefficient slightly increases with the sliding distance until the experiment ends.

According to the above results of the friction coefficient and the data in Table 1, it is shown that as the Si content gradually increases, the friction coefficient of the material with a higher percentage of Si decreases.

### 3.2. Wear Loss

**Figure 8:**
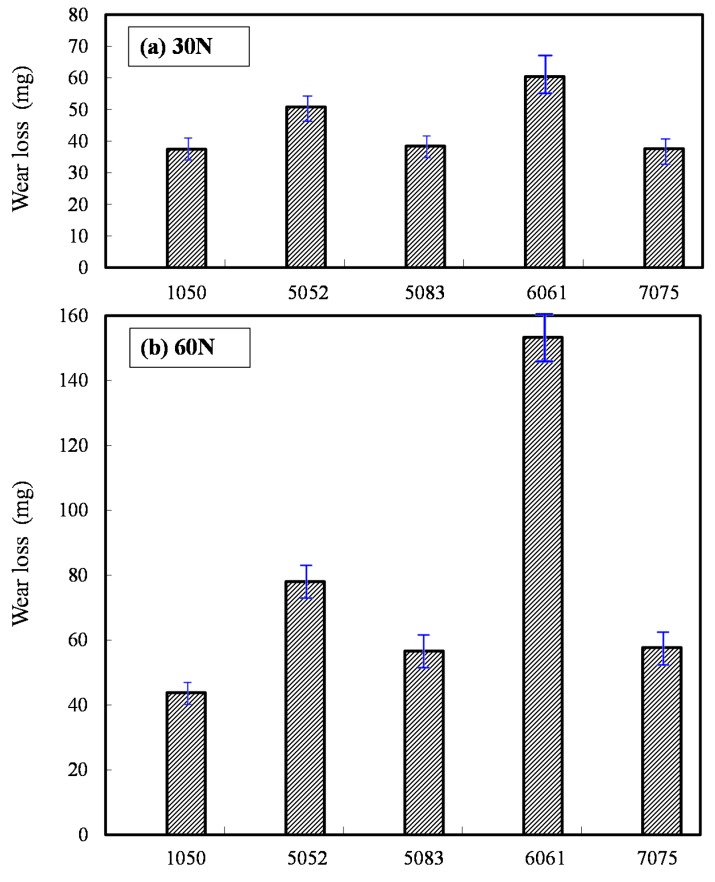
Average wear loss of the five alloys: (**a**) 30 N; (**b**) 60 N.

Three reproducibility experiments were conducted. The results of the average wear loss are shown in Figure 8. As shown in Figure 8a of 30 N, the average wear loss of 1050 aluminum alloy is 37 mg; 51 mg for 5052 aluminum alloy; 38 mg for 5083 aluminum alloy; 60 mg for 6061 aluminum alloy; and 38 mg for 7075 aluminum alloy. In Figure 8b of 60 N, the average wear loss of 1050 aluminum alloy is 44 mg, 78 mg for 5052 aluminum alloy; 57 mg for 5083 aluminum alloy; 153 mg for 6061 aluminum alloy; and 58 mg for 7075 aluminum alloy. Hence, the average wear losses are proportional to the normal loads. This indicates that the wear modes are regular. Moreover, the average wear loss of 6061 aluminum alloy is especially larger. Compared to the data in Table 1, the higher the contents of iron and copper, the more materials are removed, representing better machinability. Since the Fe/Fe and Cu/Fe pairs are compatible, this indicates that the wear losses for the compatible doping elements are larger [18]. On the other hand, it is worth noting that the wear loss of 5083 is quite small. This indicates that a higher content of Mn or Mg is harmless to the wear loss. Namely, if the pairs are incompatible, the wear losses are smaller [18]. As aluminum alloys are relatively soft and have a lower melting point, the wear particles produced in the friction process easily deviate from the contact interfaces, and the high temperature due to frictional heat results in material transfer. Therefore, optical microscopy of the wear track and SEM observation of the wear particle are both required for observing the wear mechanisms.

### 3.3. Optical Microscopy of the Wear Track 

**Figure 9:**
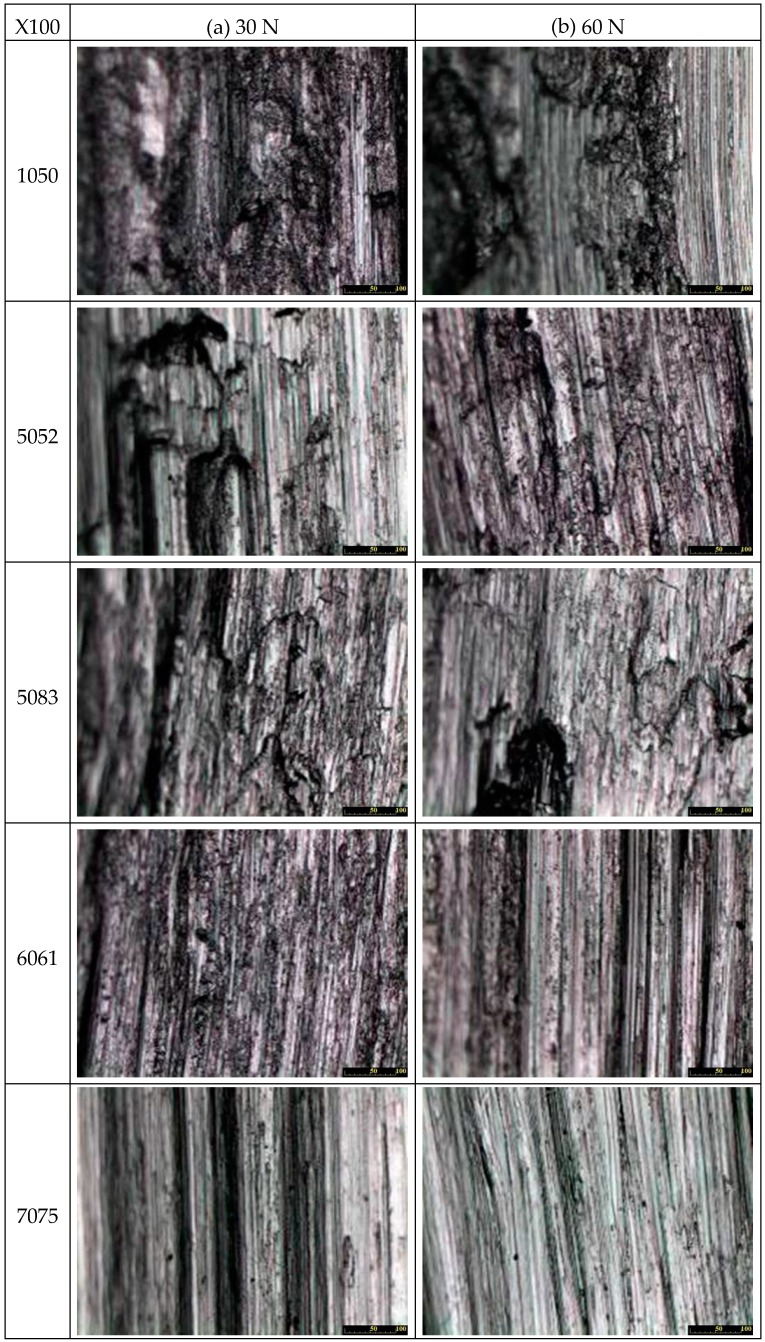
Optical microscopy of the wear track of the five alloys: (**a**) 30 N; (**b**) 60 N.

Figure 9 shows that the wear surface of the 1050 aluminum alloy presents rough wavy scratching at different widths, meaning that the vibration is relatively violent for the SUJ2 ball sliding against this material, i.e., there is significant material transfer and deformation during the friction process. As a result, many materials are extruded and adhere to areas outside the wear marks. This can be used to demonstrate that while the material is soft, the wear loss is not the largest.

The wear scratching of 5052 aluminum alloy is similar to the case of 1050 aluminum alloy, the surfaces of which present rough and wavy scratching in different widths, the vibration is slightly violent and there is some material transfer and deformation during the friction process. Thus, part of the materials that are extruded adhere to areas outside the wear marks. Moreover, it is believed that high temperatures result in material transfer and deformation during the friction process of 1050 and 5052 aluminum alloys, and the friction scratching presents many crash and melting patterns.

For 5083 aluminum alloy, the friction marks are narrower but deeper than for 1050 or 5083 aluminum alloys. This means that the wear mechanism of 5083 aluminum alloy is quite different from that of 1050 or 5083 aluminum alloys.

The wear surface of 6061 aluminum alloy is wide but with smooth friction tracks. Since the 7075 alloy sample has the highest hardness, it is to be expected that the scratched wear surface is the smoothest and the friction tracks are the narrowest. Moreover, the scratched wear surfaces of 6061 and 7075 aluminum alloys look quite smooth. These results match the previous results of wear loss.

### 3.4. SEM Observation of Wear Particle

**Figure 10:**
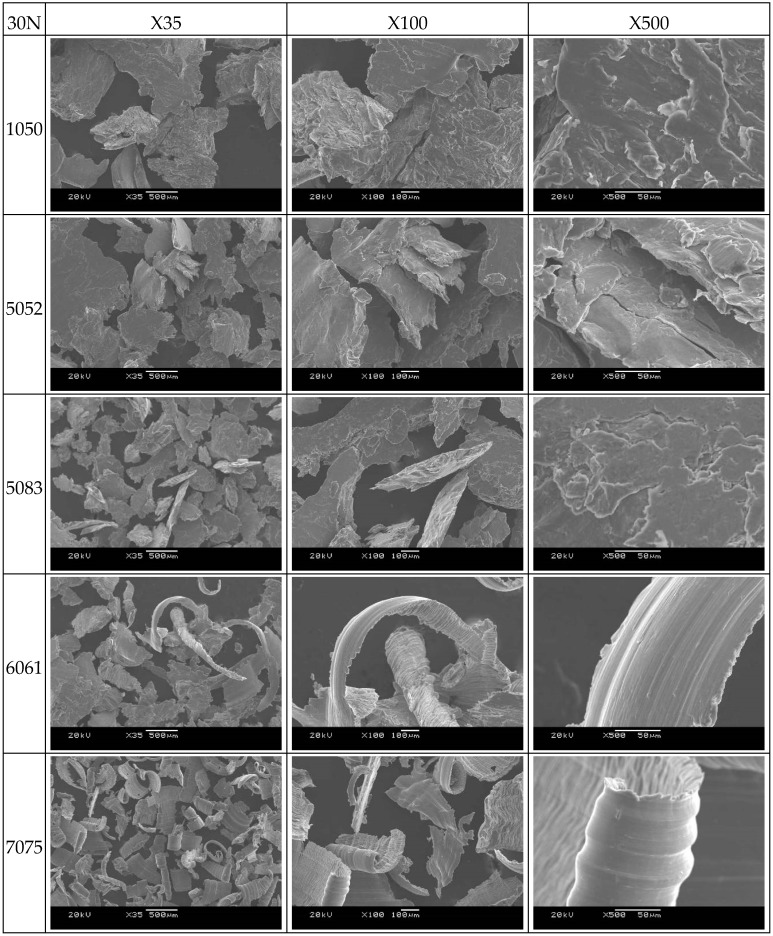
SEM of wear particle of the five alloys.

Figure 10 shows that the wear particles of 1050 and 5052 aluminum alloys are flake-like and laminated; it can be reasonable explained by the fact that these materials are relatively soft and have a lower melting point, thus resulting in deformation and material transfer due to high temperature during the friction process. A large number of wear particles of large size can be found. The large wear particles are caused by the junction growth during the adhesive wear. Therefore, in these experiments, the friction coefficient is larger than the other cases due to the action of the junction growth [29].

Most of the wear particles of 5083 aluminum alloy materials are similar to those of 1050 and 5052 aluminum alloy materials. Moreover, it is worth noting that some thick ridge-like particles are observed and the fracture mechanism of the wedge can be revealed. The wedge growth by plowing is quite significant. It is believed that the thick ridge-like particles result from the adhesive transfer of the wedge [30].

The 6061 and 7075 aluminum alloy materials produce strip-shaped wear particles. Several examples of ploughed particles are shown. In these two cases, ploughing is comparatively significant and large. It is believed that the ploughing wear particles are caused by grooving wear during the wear test [31].

### 3.5. Frictional Models and Wear Mechanisms

**Figure 11:**
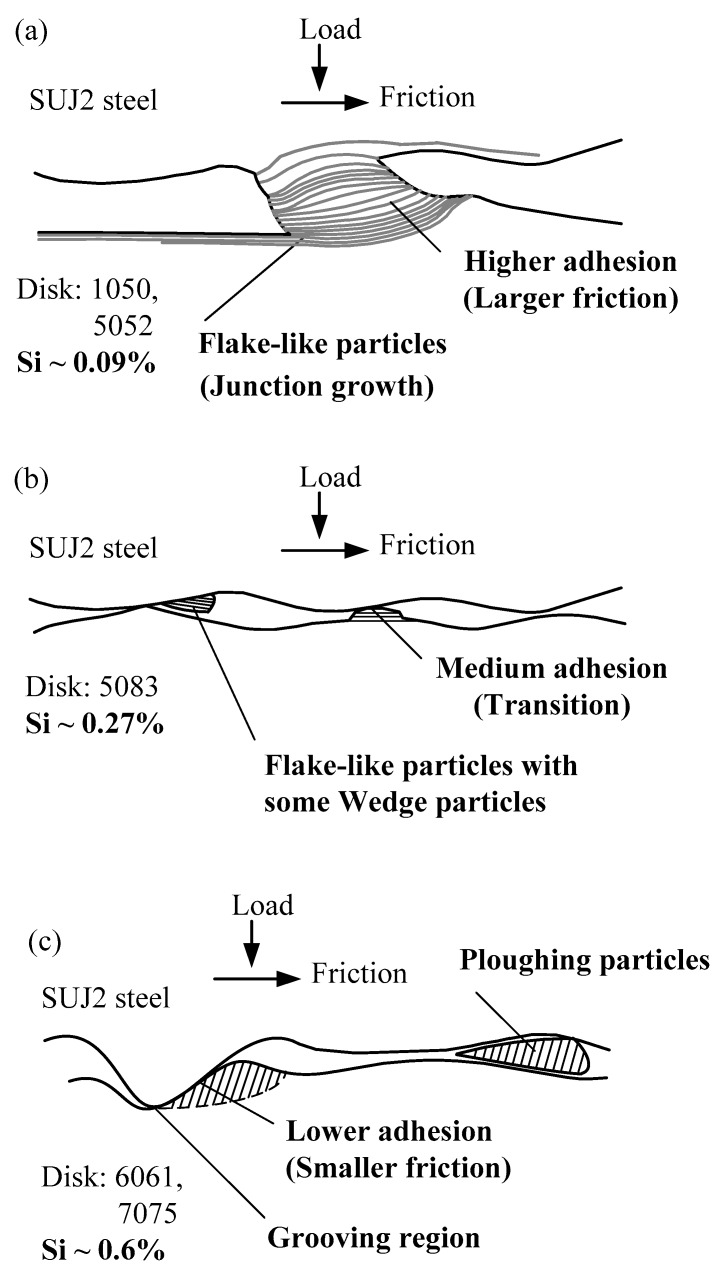
Frictional models and wear mechanisms of SUJ2 steel sliding against Al–Si alloys: (**a**) Low content of silicon: 1050 and 5052 aluminum alloys; (**b**) Medium content of silicon: 5083 aluminum alloy; (**c**) High content of silicon: 6061 and 7075 aluminum alloys.

By considering all of the above experimental results as well as the previous studies by the authors [29,30,31], three frictional models and wear mechanisms that describe the effects of the content of doping elements on the friction and wear of SUJ2 steel sliding against aluminum alloys are proposed. It is observed from Figure 11a that the flake-like particles resulted from the higher adhesion at the junction growth region for the low content of silicon (only 0.09%): 1050 and 5052 aluminum alloys. Hence, more friction is caused. Figure 11b shows that medium adhesion is present at the interfaces for the medium content of silicon (0.27%): 5083 aluminum alloy. The flake-like particles with some wedge particles were developed from both the junction growth and the adhesive transfer of the wedge. Figure 11c shows that the relatively large ploughing particles were caused by the grooving wear mechanism for the high content of silicon (~0.6%): 6061 and 7075 aluminum alloys. Hence, less friction is caused due to the lower adhesion. These results correspond to the previous friction coefficient responses. Wear mechanisms change as the silicon content increases, from the junction growth to the wedge particles, and even to the ploughing particles.

## 4. Conclusions

From the variations of the friction coefficient with optical microscopy and SEM observations of the wear, the following conclusions have been drawn:

1. The materials with a higher Si content percentage show lower friction. Generally, the friction coefficient decreases with increases in the Si content, and the variation of the friction coefficient is milder.
2. The 6061 aluminum alloy has severe ploughing and wear particles. The wear losses for the compatible doping elements are larger. Therefore, the higher the contents of iron and copper, the more materials are removed, representing better machinability.
3. Three frictional models and wear mechanisms that describe the effects of the content of doping elements on the friction and wear of SUJ2 steel sliding against aluminum alloys are proposed. The wear mechanisms change as the silicon content increases, from the junction growth to the wedge and the ploughing particles.
4. Based on the above three models, better choices of aluminum alloys with regards to friction and wear can be made. These results have great practical importance for the precision machinery industry.

## Figures and Tables

**Table 1:** Determination of the content of the five disks (wt %).

Aluminum Alloys	Si	Fe	Cu	Mn	Mg
1050	0.09	0.23	0.01	0.01	0.03
5052	0.09	0.26	0.02	0.05	2.50
5083	0.27	0.38	0.09	0.59	4.70
6061	0.58	0.62	0.26	0.06	1.07
7075	0.60	0.20	0.16	0.07	1.00
